# Cutaneous Myiasis in Traveler Returning from Ethiopia

**DOI:** 10.3201/eid1712.111062

**Published:** 2011-12

**Authors:** Paul Hannam, Krishna Khairnar, James Downey, Jeff Powis, Filip Ralevski, Dylan R. Pillai

**Affiliations:** Author affiliations: Toronto East General Hospital, Toronto, Ontario, Canada (P. Hannam, J. Downey, J. Powis);; Ontario Agency for Health Protection and Promotion, Toronto (K. Khairnar, F. Ralevski, D.R. Pillai);; University of Toronto, Toronto (D.R. Pillai)

**Keywords:** cutaneous myiasis, human, traveler, Canada, flies, zoonoses, Lund’s fly, Cordylobia rodhaini, parasites, Ethiopia

**To the Editor:** Myiasis is an infestation of human tissue by the larval stage of flies of the order Diptera. There are 3 clinical manifestations of myiasis: localized furuncular myiasis typically caused by *Dermatobia hominis*, *Cordylobia anthropophaga*, *Wohlfahrtia vigil*, and *Cuterebra* spp.; creeping dermal myiasis caused by *Gasterophilus* spp. and *Hypoderma* spp.; and wound and body cavity myiasis caused by *Cochliomyia hominivorax*, *Chrysomya bezziana*, and *Wohlfahrtia magnifica* (*1*). The Tumbu fly (*C. anthropophaga*) and the human botfly (*D. hominis*) are the most common vectors for myiasis in Africa and the tropical Western Hemisphere, respectively (*2*). The genus *Cordylobia* also contains 2 less common species (*C. ruandae* and *C. rodhaini*) (*3*). Infection with *C. rodhaini* (Lund’s fly) is less common.

A review of the literature showed only 7 reports of *C. rodhani* myiasis in travelers from countries such as Australia (*3*), Italy (*4*), Canada (*5*), France (*6*), and Israel (*7*). All travelers were infested after travel to eastern and western regions of central Africa. In humans, the skin lesion starts as a painful red papule that gradually enlarges and develops into a furuncle. Typically, the center of the lesion has an opening, through which the larva breaths and discharges its waste products. Cutaneous myiasis is usually an uncomplicated and self-limiting disease. The flies have adapted to tropical environments, and spread to areas in which this disease is not endemic is unlikely.

In the emergency department, cellulitis or furuncular lesions are common with a broad differential diagnosis. With the introduction of bedside ultrasonography in the emergency department, ultrasonographic evaluation of soft tissue infections is more accurate than clinical examination in detecting abscesses (*8**,**9*). Ultrasonographic examination of soft tissue infections enables more accurate localization of an associated abscess and the potential to more specifically identify etiology such as a foreign body (*10*). We report a rare case of cutaneous myiasis caused by *C. rodhaini* larvae in a traveler returning from tropical Africa.

The patient was a 26-year-old woman who came to the emergency department at Toronto East General Hospital with a 10-day history of a painful red lesion on her left upper arm. She had first assumed it to be an insect bite, but during the preceding few days the swelling had greatly increased. She had no constitutional symptoms other than a persisting mild cough for which she had taken a 5-day course of amoxicillin ≈2 weeks before coming to the hospital. Her medical history was noncontributory. She reported that 7 days earlier she had returned from a 1-month trip to Ethiopia. During her stay in Ethiopia, she had been primarily in rural areas but did not report contact with sick persons. Her vital signs were normal.

Physical examination showed a 2.5-cm^2^ erythematous area on the lateral aspect of the upper arm (Figure, panel A). There was a 1-mm central punctum and local tenderness. Discharge, streaking, or proximal adenopathy were not present. Other results of the examination were noncontributory. Results of a complete blood count and liver function tests were within reference ranges. Results of a chest radiographic were normal.

**Figure:**
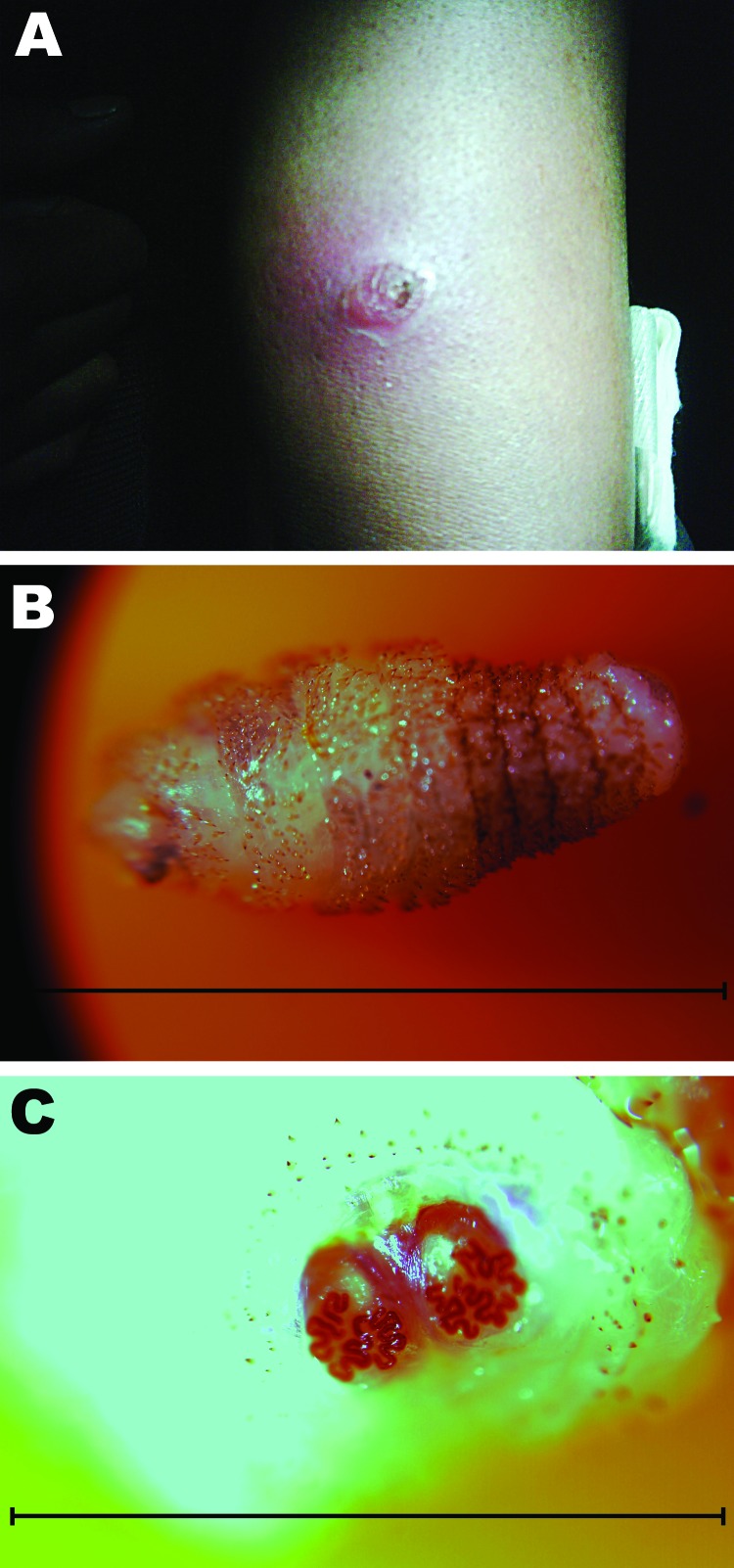
A) Lateral aspect of the upper arm of a 26-year-old woman showing cutaneous myiasis and an erythematous lesion 2.5 cm in diameter, Canada. B) *Cordylobia rodhaini* larva (length ≈1 cm) isolated from the erythematous lesion. Scale bar = 10 mm. C) Characteristic posterior spiracles of a *C. rodhaini* larva. Scale bar = 3 mm.

Bedside ultrasonography was performed to assess possible abscess. During ultrasonography, the patient reported a biting sensation and increased pain in the area of the lesion. Ultrasonographic images of the lesion showed an area of spontaneous movement just below the skin, suggestive of cutaneous myiasis (Video).

**Video:**
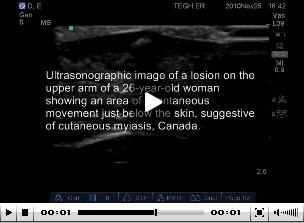
Ultrasonographic image of a lesion on the upper arm of a 26-year-old woman who returned to Canada from Ethiopia, showing an area of spontaneous movement just below the skin, suggestive of cutaneous myiasis.

Direct Video Link: http://streaming.cdc.gov/vod.php?id=23c872c98cac849c2d2d01ae4229456620111201171809133

Treatment for myiasis can be conservative or surgical. Surgical treatment consists of mechanical removal of the larva. After consultation with infectious disease specialists, we covered the lesion with standard lubricating jelly and Op Site Flexfix transparent adhesive (Smith and Nephew, St. Laurent, Quebec, Canada) to obtain a seal. Approximately 45 minutes later, a 1-cm, white–yellow larvae emerged from the area and was removed intact with the dressing (Figure, panel B). *C. rodhaini* was identified by its characteristic posterior spiracles and the pattern of the larvae (Figure, panel C). Another occlusive dressing was applied before patient discharge. At follow-up 4 days later, the lesion was no longer symptomatic and the patient refused further treatment.

Physicians should consider myiasis in patients who have a furuncular lesion after returning from tropical countries. Bedside ultrasonography rapidly confirmed the diagnosis of myiasis, enabling immediate and appropriate treatment. Travelers should be aware of this potential infestation with the less common Lund’s fly and not only avoid direct contact with clothes left outside but also avoid direct contact with infested material (*5*).
